# Sepsis-associated gastrointestinal dysfunction: neuroimmune mechanisms, gut–brain axis disruption, and therapeutic potential of vagus nerve–targeted neuromodulation

**DOI:** 10.3389/fmed.2026.1841517

**Published:** 2026-05-28

**Authors:** Zhiyang Wu, Bo Yao, Jian Yu

**Affiliations:** 1 Department of Critical Care Medicine, The Second Affiliated Hospital of Dalian Medical University, Dalian, Liaoning, China; 2 Department of Critical Care Medicine, Qilu Hospital (Qingdao), Cheeloo College of Medicine, Shandong University, Qingdao, Shandong, China

**Keywords:** feeding intolerance, gut–brain axis, intestinal barrier dysfunction, neuroimmune interaction, transcutaneous auricular vagus nerve stimulation, vagus nerve

## Abstract

Sepsis-associated gastrointestinal dysfunction (SAGD) is a frequent complication in critically ill patients, marked by impaired motility, feeding intolerance, and disruption of the intestinal barrier, and is linked to poor clinical outcomes. Growing evidence indicates that SAGD arises from the interaction between systemic inflammation and neuroimmune dysregulation. Pro-inflammatory cytokines such as tumor necrosis factor, interleukin-1β, and interleukin-6 promote epithelial injury, disturb tight junction integrity, and impair neuromuscular function, while microcirculatory disturbances further aggravate tissue hypoxia and barrier breakdown. At the same time, alterations in the gut–brain axis and autonomic imbalance compromise gastrointestinal motility and disrupt the cholinergic anti-inflammatory pathway, suggesting that SAGD represents a coordinated inflammatory–barrier–neuroimmune disorder. Current management remains mainly supportive and does not target these underlying processes. As a central element of the gut–brain axis, the vagus nerve plays a key role in regulating both immune responses and gastrointestinal function. Neuromodulation approaches aimed at vagal pathways—especially transcutaneous auricular vagus nerve stimulation (taVNS)—have therefore attracted attention as non-invasive strategies to influence inflammation and autonomic balance. Although direct clinical evidence in SAGD is still limited, the biological rationale supports further exploration.

## 1 Introduction

Sepsis is a life-threatening condition characterized by a dysregulated host response to infection and remains a major cause of morbidity and mortality worldwide (1–3). Among its multiple organ manifestations, sepsis-associated gastrointestinal dysfunction (SAGD) has been increasingly recognized as a frequent and clinically relevant complication in critically ill patients (4–6). SAGD typically presents with gastrointestinal dysfunction, including impaired motility, feeding intolerance, abdominal distension, and disruption of intestinal barrier integrity, and is associated with delayed initiation and reduced tolerance of enteral nutrition, prolonged intensive care unit stay, and poorer clinical outcomes (4–8). Importantly, its temporal progression beyond the acute phase remains poorly defined. Despite its clinical relevance, SAGD is still underrecognized and insufficiently characterized in both clinical practice and research settings.

Clinically, SAGD is most commonly recognized during the acute and subacute phases of sepsis, when gastrointestinal dysfunction becomes evident. While gastrointestinal function may partially recover with resolution of systemic inflammation, sepsis-related disturbances—including persistent low-grade inflammation, autonomic dysregulation, epithelial injury, and gut microbiota alterations—may extend beyond the acute phase and contribute to prolonged gastrointestinal dysfunction in some survivors. Compared with well-characterized post-sepsis sequelae such as cognitive impairment and immune dysregulation, the persistence and clinical significance of gastrointestinal dysfunction after sepsis remain underexplored.

A growing body of evidence indicates that SAGD is not simply a secondary consequence of critical illness but rather a complex syndrome shaped by the interplay among systemic inflammation, intestinal barrier dysfunction, microcirculatory impairment, and neuroimmune dysregulation, including disruption of the gut–brain axis (9–12). These interconnected processes are increasingly recognized at a systems level, although many studies have examined them in isolation, and an integrated framework is still evolving (9–12). Accordingly, SAGD is better viewed as an integrated inflammatory–barrier–neuroimmune disorder rather than an isolated gastrointestinal complication. Detailed molecular and cellular mechanisms underlying these processes are discussed in subsequent sections (9–12). However, direct clinical evidence linking these interconnected mechanisms to SAGD-specific outcomes remains limited (9, 12).

The gastrointestinal tract occupies a central position at the interface between host immunity and the external environment and has been proposed as a potential amplifier of systemic inflammation during sepsis (9–12). Loss of intestinal barrier integrity may allow translocation of bacteria, endotoxins, and damage-associated molecular patterns into the systemic circulation, thereby perpetuating immune activation and contributing to multiple organ dysfunction (9–11). Meanwhile, gastrointestinal dysmotility restricts the delivery and absorption of enteral nutrition, exacerbating metabolic stress and impairing mucosal immunity, and may alter gut microbiota composition (5–10). These bidirectional interactions highlight the central role of the gut in sepsis progression and underscore the need for therapeutic strategies that address both immune and neural regulatory pathways (9–13).

Current management of SAGD remains largely supportive and symptom-oriented (5–8). Pharmacological interventions, such as prokinetic agents, are widely used to enhance gastrointestinal motility, while nutritional strategies, including early enteral nutrition, aim to preserve gut integrity and immune function (7, 8). However, these approaches show inconsistent efficacy in septic patients and do not adequately target the underlying mechanisms of inflammation, barrier dysfunction, and neuroimmune imbalance (5–8). In addition, their effectiveness may be constrained by impaired enteric nervous system function and altered smooth muscle responsiveness in the septic state (11, 12). Notably, current treatment paradigms largely overlook the role of the autonomic nervous system and gut–brain axis, which are increasingly recognized as key regulators of gastrointestinal function and systemic inflammation (11–17), creating a gap between complex pathophysiology and limited therapeutic targeting (5–8, 14–17).

The vagus nerve represents a central neuroimmune interface within the gut–brain axis, linking central neural circuits with peripheral immune responses and gastrointestinal function (14, 15, 17). By coordinating immune and enteric neural signaling, vagal pathways may influence inflammatory activity, gastrointestinal motility, and barrier homeostasis (11, 14, 15). Altered vagal modulation has been associated with disease severity and impaired heart rate variability in sepsis (13). While altered or reduced vagal modulation is often reported during the acute phase, other studies suggest that vagal responses may be preserved or vary across disease stages, reflecting a dynamic imbalance in sympathovagal regulation rather than a uniform reduction in vagal tone (13). This more nuanced understanding has prompted increasing interest in neuromodulation strategies targeting vagal pathways as a means to restore neuroimmune homeostasis.

Among these approaches, transcutaneous auricular vagus nerve stimulation (taVNS) has emerged as a non-invasive and clinically feasible technique capable of modulating autonomic activity and engaging central regulatory circuits. By acting on both afferent and efferent pathways, taVNS may influence downstream neuroimmune responses, attenuate systemic inflammation, and potentially improve gastrointestinal function (17, 18). Experimental studies, along with evidence from related clinical conditions such as functional gastrointestinal disorders and inflammatory diseases, suggest that taVNS can affect both immune signaling and gastrointestinal motility (17–19). However, direct clinical evidence in patients with SAGD remains limited, and key questions regarding optimal stimulation protocols, patient selection, and clinically meaningful endpoints remain unresolved (17–19).

While prior reviews have addressed sepsis-related intestinal injury, gut–brain axis signaling, or vagus nerve stimulation separately, a focused synthesis linking SAGD pathophysiology with vagus nerve–targeted neuromodulation remains lacking. In this context, this review aims to integrate current evidence on inflammatory and neuroimmune mechanisms, examine the role of gut–brain axis disruption and autonomic dysregulation, and critically evaluate the translational potential and limitations of vagus nerve–targeted neuromodulation, particularly taVNS, with attention to the distinction between mechanistic plausibility, indirect clinical evidence, and direct SAGD-specific validation (9, 12, 17–19).

## 2 Pathophysiology of sepsis-associated gastrointestinal dysfunction

### 2.1 Inflammatory and neuroimmune mechanisms of gastrointestinal dysfunction

Sepsis-associated gastrointestinal dysfunction (SAGD) develops through a complex and closely interconnected interaction between systemic inflammation and neuroimmune dysregulation (11, 20–22). Excessive inflammatory responses, reflected by elevated circulating levels of tumor necrosis factor (TNF), interleukin-1β (IL-1β), and interleukin-6 (IL-6), are considered a major driver of intestinal injury (20, 23–28). These cytokines disrupt epithelial homeostasis by promoting epithelial injury, apoptosis, and barrier instability, thereby linking systemic inflammation with intestinal dysfunction (20, 23–28). At the same time, activation of intracellular inflammatory signaling cascades—particularly nuclear factor-κB (NF-κB) and mitogen-activated protein kinase (MAPK) pathways—further enhances cytokine production, sustains mucosal inflammation, and contributes to ongoing barrier dysfunction (20, 24, 26–28). In addition, oxidative stress and mitochondrial dysfunction driven by inflammatory mediators may further compromise epithelial integrity and cellular energy metabolism (20, 23–28).

Beyond direct epithelial injury, inflammatory mediators also exert marked effects on gastrointestinal neuromuscular function (11, 21, 29–32). Cytokines can impair smooth muscle contractility by disturbing intracellular calcium homeostasis and excitation–contraction coupling, thereby reducing coordinated peristalsis (11, 21, 31, 32). At the level of the enteric nervous system (ENS), inflammatory signaling influences neurotransmitter release and receptor sensitivity, including pathways involving acetylcholine, nitric oxide, and vasoactive intestinal peptide (11, 21). Structural and functional alterations of the ENS—such as neuronal apoptosis, enteric glial cell dysfunction, and impaired neurotrophic signaling—further limit the integration of neural circuits required for coordinated gastrointestinal motility (11, 21, 29, 30). Disruption of interstitial cells of Cajal, which act as electrical pacemakers of intestinal peristalsis, may additionally contribute to dysrhythmic motility patterns and delayed intestinal transit (31, 32).

Autonomic dysregulation represents a key interface linking inflammation with neural dysfunction in SAGD (11, 13, 21, 22). Altered vagal modulation, often accompanied by context-dependent sympathovagal imbalance during sepsis, may impair gastrointestinal motility and disrupt coordinated neuroimmune signaling (13, 22, 33, 34). Functionally, this dysregulation contributes to impaired gut–brain communication and amplification of inflammatory responses (11, 21, 22). The detailed neuroimmune mechanisms underlying vagal modulation are discussed in section 4.1. Taken together, these findings support the view that gastrointestinal dysfunction in sepsis reflects an integrated inflammatory–neural disturbance rather than isolated organ pathology (11, 13, 20–22).

### 2.2 Intestinal barrier dysfunction and microcirculatory impairment

The intestinal barrier plays a fundamental role in maintaining host–microbial homeostasis, and its disruption represents a central feature of SAGD. Sepsis-induced barrier dysfunction is characterized by increased epithelial permeability, loss of tight junction integrity, and apoptosis of intestinal epithelial cells (35–37). At the molecular level, inflammatory mediators reduce the expression and alter the localization of tight junction proteins such as occludin, claudins, and ZO-1 (35, 38, 39), while activation of myosin light chain kinase (MLCK) promotes cytoskeletal contraction and increases paracellular permeability (40–43). In addition, changes in epithelial turnover, impaired stem cell function, and disruption of epithelial restitution may further hinder barrier regeneration (36, 44). The mucus layer, which serves as an important physical and biochemical barrier, is also disrupted during sepsis, accompanied by decreased secretion of mucins and antimicrobial peptides, thereby weakening mucosal defense mechanisms (35, 36).

Microcirculatory dysfunction further aggravates intestinal injury and represents a key contributor to barrier failure (37, 45, 46). Reduced splanchnic perfusion during sepsis leads to tissue hypoxia, mitochondrial dysfunction, and excessive generation of reactive oxygen species (ROS), which together promote epithelial injury and inflammatory activation. These processes are further amplified by ischemia–reperfusion injury and endothelial dysfunction, creating a self-reinforcing cycle of impaired perfusion, oxidative stress, and mucosal damage (45–50). Importantly, restoration of systemic hemodynamic parameters does not necessarily normalize intestinal microcirculatory flow, highlighting a dissociation between global circulation and regional tissue perfusion (46, 51).

As a result of barrier disruption, translocation of bacteria (52), endotoxins, and damage-associated molecular patterns into the systemic circulation may occur, further activating innate immune responses through pattern recognition receptors such as Toll-like receptors (TLRs) (36, 37). This process sustains a self-perpetuating cycle of inflammation and organ dysfunction, supporting the view of the gut as a potential driver and amplifier of systemic inflammatory responses in sepsis (37, 52). Although the extent and clinical relevance of bacterial translocation in human sepsis remain under investigation, this mechanism provides a useful conceptual framework linking barrier failure with systemic disease progression (52).

### 2.3 Gut–brain axis disruption and autonomic dysregulation

The gut–brain axis constitutes a bidirectional communication network that integrates neural, immune, and endocrine signaling pathways and plays an important role in maintaining gastrointestinal and systemic homeostasis (14, 53–55). In sepsis, this regulatory system becomes markedly disrupted, contributing to both gastrointestinal dysfunction and dysregulated immune responses (54, 56). Communication within this axis involves vagal afferent and efferent pathways, circulating inflammatory mediators, and microbial-derived metabolites, all of which can be altered during sepsis (14, 54, 55, 57, 58).

Autonomic nervous system dysregulation is a defining feature of sepsis and reflects a context-dependent imbalance in sympathovagal activity rather than a uniform reduction in vagal tone (13, 59, 60). Functionally, this dysregulation contributes to impaired gastrointestinal motility, delayed gastric emptying, and disrupted gut–brain communication. These alterations should be understood as part of a broader neuroimmune disturbance, the detailed mechanisms of which are discussed in section 4.1.

Beyond peripheral autonomic dysregulation, changes in central autonomic control and enteric nervous system signaling further disturb coordination between the brain and the gastrointestinal tract (14, 53, 54, 57). Neuroinflammatory processes within central regulatory circuits may impair vagal output and shift autonomic setpoints (14, 54, 57, 61). Emerging evidence also suggests that sepsis-associated alterations in gut microbiota composition and function may influence gut–brain communication through immune, metabolic, and neuroactive pathways, including the production of short-chain fatty acids and other microbial metabolites. Although these mechanisms are not yet fully defined, they add another layer of complexity to the pathophysiology of SAGD and may partly account for inter-individual variability in clinical presentation (54–56).

### 2.4 Integrated pathophysiological framework

SAGD can be viewed as the result of dynamically interacting disturbances involving inflammation, barrier integrity, microcirculation, and neuroimmune regulation (53, 62, 63). As described above, systemic inflammatory responses contribute to epithelial injury and disruption of tight junction integrity, while microvascular dysfunction further aggravates tissue hypoxia, oxidative stress, and mitochondrial injury (37, 62, 63). At the same time, autonomic imbalance together with enteric nervous system dysfunction impairs gastrointestinal motility and neural regulation, thereby worsening functional impairment (53, 63). These processes promote bacterial translocation and amplify systemic inflammation, forming a self-reinforcing cycle that contributes to multiple organ dysfunction (37, 63).

**FIGURE 1:**
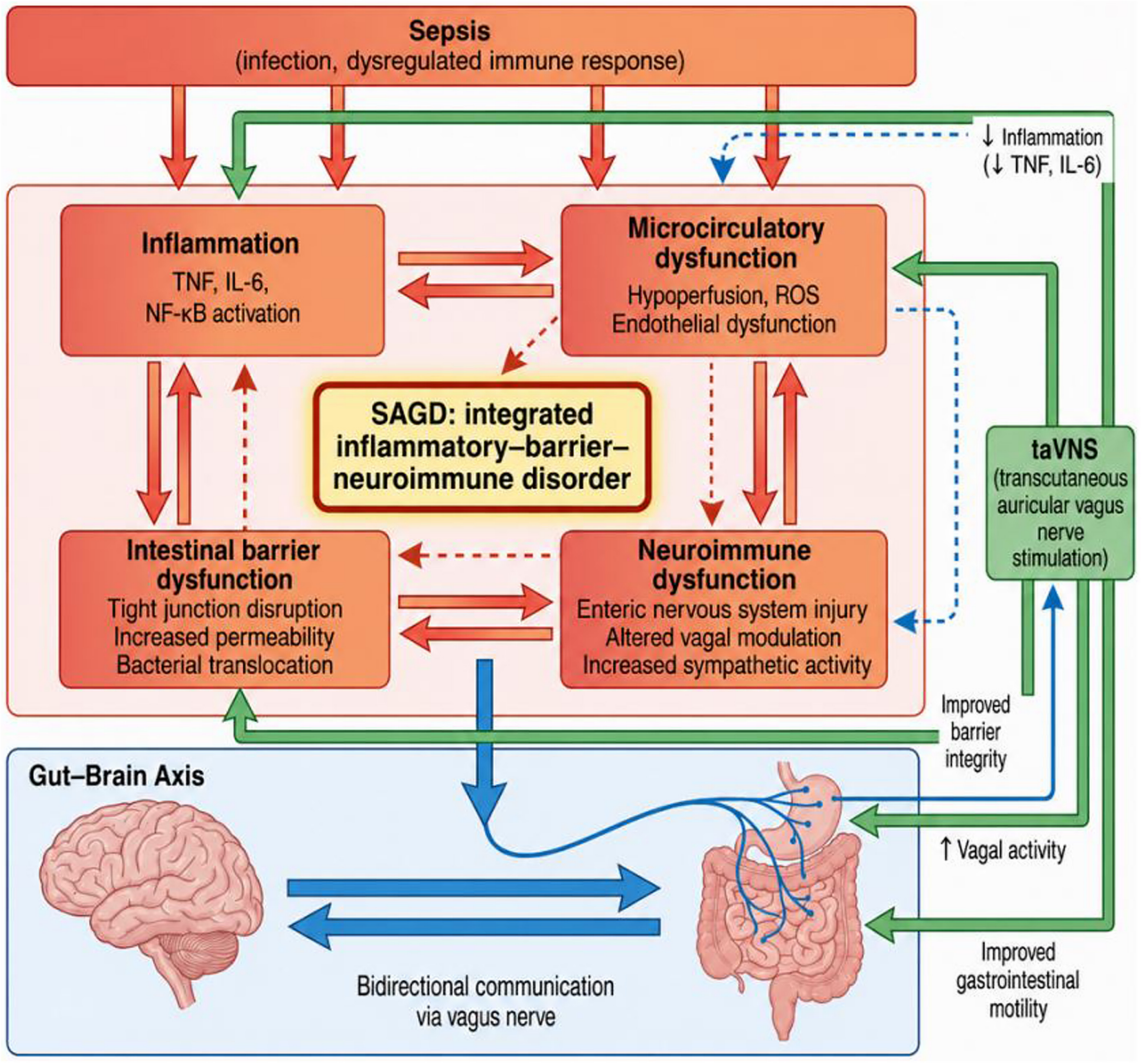
Integrated pathophysiology of SAGD and vagus nerve–mediated neuromodulation. SAGD arises from the interaction of systemic inflammation, intestinal barrier disruption, microcirculatory dysfunction, and neuroimmune dysregulation, forming a self-amplifying network. The gut–brain axis, mediated by the vagus nerve, links gastrointestinal and central nervous system function. In sepsis, altered vagal modulation and sympathovagal imbalance contribute to both inflammation and gastrointestinal dysfunction. Transcutaneous auricular vagus nerve stimulation (taVNS) may modulate this network by reducing inflammation, enhancing vagal activity, and improving gastrointestinal motility and barrier integrity. Solid arrows indicate established pathways, and dashed arrows indicate proposed mechanisms.

Importantly, these mechanisms do not operate in isolation but are closely interconnected, with inflammation aggravating neural dysfunction and neuroimmune dysregulation, in turn, further enhancing inflammatory signaling (53, 63). This integrated framework highlights the central role of the gut as both a target and an active contributor to sepsis progression (37, 63). Importantly, it underscores the need for therapeutic strategies that target both inflammatory and neuroimmune pathways, thereby providing a rationale for the intervention approaches discussed in the following section. The integrated pathophysiological framework of SAGD and its modulation by vagal pathways are illustrated in Figure 1.

## 3 Current therapeutic strategies and limitations in sepsis-associated gastrointestinal dysfunction

### 3.1 Pharmacological and prokinetic approaches: symptom-oriented interventions

Pharmacological therapies for sepsis-associated gastrointestinal dysfunction (SAGD) are mainly directed at improving gastrointestinal motility and facilitating enteral feeding, rather than addressing the underlying pathophysiological mechanisms. Prokinetic agents, including metoclopramide and erythromycin, are widely used in critically ill patients with feeding intolerance and are recommended in clinical practice guidelines (7, 8, 64). These agents enhance gastric emptying through dopaminergic antagonism or motilin receptor activation and may transiently improve feeding tolerance; however, their clinical efficacy is variable and often short-lived, particularly in the setting of persistent systemic inflammation and autonomic dysregulation characteristic of sepsis (65–68).

Several factors limit their effectiveness in this context. Tachyphylaxis, especially with erythromycin, reduces sustained therapeutic benefit, while adverse effects—including QT interval prolongation, cardiac arrhythmias, and extrapyramidal symptoms—restrict broader use (69, 70). In addition, these agents primarily target upper gastrointestinal motility and do not directly affect key mechanisms underlying SAGD, such as epithelial barrier disruption, microcirculatory dysfunction, or neuroimmune imbalance (8, 64, 68). Their efficacy may also be reduced by impaired enteric nervous system signaling and diminished smooth muscle responsiveness during sepsis, conditions in which downstream effector pathways are inherently compromised. Emerging pharmacological strategies, including ghrelin receptor agonists and serotonin receptor modulators, have been explored in experimental and early clinical settings; however, their roles in septic populations remain incompletely defined and require further validation (68). Taken together, current pharmacological approaches remain largely symptom-oriented and are insufficient to reverse the integrated pathophysiological disturbances observed in SAGD (8, 64, 68). The supporting evidence is primarily derived from clinical experience and indirect studies, with limited SAGD-specific validation.

### 3.2 Nutritional strategies: supportive but mechanistically limited

Early enteral nutrition (EEN) remains a cornerstone of supportive care in critically ill patients and is strongly recommended by international guidelines, including those from SCCM/ASPEN and ESPEN. EEN is generally considered to help preserve gut integrity, maintain mucosal immunity, and reduce infectious complications compared with parenteral nutrition (7, 8, 64, 71). From a mechanistic perspective, luminal nutrient delivery may stimulate epithelial turnover, enhance mucosal blood flow, and promote the release of gastrointestinal hormones and trophic factors that support barrier function and immune regulation. In addition, enteral feeding may help sustain gut microbiota diversity and metabolic activity, thereby indirectly influencing host–microbial interactions (71, 72).

Despite these potential benefits, feeding intolerance remains highly prevalent in patients with sepsis and gastrointestinal dysfunction, often limiting the effective delivery of enteral nutrition. Clinical manifestations such as increased gastric residual volumes, vomiting, abdominal distension, and diarrhea frequently require interruption or adjustment of feeding protocols (64, 68, 73). These challenges largely reflect underlying dysmotility, barrier dysfunction, and neuroimmune dysregulation, which are not directly corrected by nutritional interventions. In particular, impaired vagal signaling together with enteric nervous system dysfunction may restrict the capacity of enteral feeding to restore coordinated gastrointestinal motility and digestive function (68, 71).

Alternative strategies, such as post-pyloric feeding, have been proposed to bypass gastric dysmotility; however, evidence supporting their superiority over gastric feeding remains inconsistent, and their use may be constrained by technical complexity and patient-specific factors (64, 72, 74). Moreover, although nutritional support is essential for preventing malnutrition and supporting immune function, it does not directly influence systemic inflammatory signaling, autonomic imbalance, or gut–brain axis disruption. Thus, nutritional strategies are best regarded as supportive measures that help preserve gut homeostasis but do not address the core mechanisms driving SAGD (8, 71, 72). Their benefits are supported mainly by indirect clinical evidence rather than direct SAGD-specific validation.

### 3.3 Supportive and microbiota-targeted therapies: indirect modulation of gut function

Supportive management, including fluid resuscitation, hemodynamic optimization, and reduction of sedative and opioid exposure, remains fundamental in the treatment of sepsis and may indirectly affect gastrointestinal function (4, 62). Adequate splanchnic perfusion is essential for maintaining intestinal integrity, and restoration of macro-hemodynamic stability may partly alleviate epithelial injury and barrier dysfunction. However, sepsis is frequently accompanied by persistent microcirculatory heterogeneity, endothelial dysfunction, and regional perfusion deficits, which are not always corrected by global hemodynamic optimization. This mismatch underscores the limitation of systemic resuscitation strategies in restoring intestinal microcirculatory function and highlights the importance of regional perfusion dynamics (62, 75).

Sedatives and opioids, which are commonly used in critically ill patients, may further impair gastrointestinal motility through both central and peripheral mechanisms. Efforts to reduce their use, when clinically appropriate, may therefore help improve gastrointestinal function, although such strategies need to be balanced against the requirement for adequate analgesia and sedation (4). Modulation of the gut microbiota has emerged as a potential therapeutic approach, given the association between sepsis and intestinal dysbiosis. Sepsis is often associated with reduced microbial diversity, loss of commensal bacteria, and overgrowth of opportunistic pathogens, which may further compromise barrier integrity and immune homeostasis (56, 76). Interventions such as probiotics, prebiotics, synbiotics, and selective digestive decontamination have been explored to restore microbial balance and reduce infection risk. However, clinical evidence remains heterogeneous, with variable effects on clinical outcomes, and safety concerns—particularly the risk of bacteremia or fungemia in immunocompromised patients—limit broader use (77–79).

Importantly, both supportive and microbiota-targeted interventions mainly exert indirect effects on gastrointestinal function and do not directly address upstream drivers of SAGD, including systemic inflammation, autonomic dysfunction, and impaired neuroimmune regulation. The available evidence remains heterogeneous and largely indirect, with limited confirmation in SAGD-specific clinical settings (56, 76).

### 3.4 Limitations of current therapies and the need for mechanism-based interventions

Despite the availability of multiple therapeutic strategies, the management of SAGD remains challenging, and clinical outcomes are often less than optimal. Most current interventions are supportive or symptom-oriented and do not sufficiently address the core pathophysiological mechanisms identified in sepsis, including systemic inflammation, intestinal barrier disruption, microcirculatory dysfunction, and neuroimmune dysregulation. This misalignment between therapeutic targets and disease mechanisms likely contributes to the limited and inconsistent clinical efficacy observed in current practice (37, 62, 80).

Notably, relatively little attention has been paid to the role of the autonomic nervous system and gut–brain axis in existing treatment paradigms. Current approaches tend to overlook the bidirectional interactions between neural, immune, and gastrointestinal systems, which are increasingly recognized as central to disease progression (37, 53). As a consequence, therapies that improve a single aspect of gastrointestinal dysfunction—such as motility or nutrient delivery—may not translate into meaningful improvements in overall gastrointestinal function or systemic outcomes (37, 80).

In addition, heterogeneity among septic patients—including differences in disease severity, timing of intervention, hemodynamic status, and extent of gastrointestinal involvement—further complicates treatment responses and may contribute to variability in clinical outcomes (62, 80). The absence of standardized definitions, biomarkers, and clinically relevant endpoints for SAGD also limits the ability to evaluate and compare therapeutic strategies across studies (37, 80).

Taken together, these limitations reflect a clear mismatch between current supportive or symptom-oriented therapies and the integrated pathophysiology of SAGD (37, 53, 80). This gap highlights the need for mechanism-based interventions capable of modulating both inflammatory and neuroimmune pathways. In this context, emerging neuromodulation strategies targeting vagal pathways provide a biologically plausible and mechanism-based alternative, which will be discussed in the following section. The comparison between current standard treatments and emerging neuromodulation therapies is summarized in Table 1.

**TABLE 1:** A comparison of current standard therapies and emerging neuromodulation approaches for SAGD.

Strategy	Target	Mechanism	Potential benefit	Key limitations	Evidence level
Prokinetics (metoclopramide, erythromycin)	Motility	Dopamine antagonism; motilin receptor activation	↑ gastric emptying; improved feeding tolerance	Tachyphylaxis; QT prolongation; no effect on inflammation/barrier	Clinical use; limited SAGD-specific data
Enteral nutrition	Gut integrity; nutrition	Luminal nutrients; mucosal support	Maintains gut integrity; supports immunity	Limited by feeding intolerance; not mechanism-targeted	Guideline-supported supportive care
Post-pyloric feeding	Nutrient delivery	Bypasses gastric dysfunction	Improved feeding delivery	Technical complexity; no effect on underlying mechanisms	Clinical evidence (non-SAGD specific)
Hemodynamic optimization	Perfusion	Fluid resuscitation; vasopressors	Supports systemic perfusion	Does not restore microcirculation	Standard sepsis care
Microbiota-targeted therapy	Dysbiosis	Modulates microbiota and immune signaling	Potential immune/barrier effects	Heterogeneous evidence; safety concerns	Mixed clinical evidence
VNS (implantable)	Neuroimmune regulation	Vagal anti-inflammatory pathways	↓ cytokines; improved survival (models)	Invasive; not feasible in ICU sepsis	Preclinical evidence
taVNS	Neuroimmune; autonomic	Auricular vagal stimulation; central modulation	Non-invasive; may modulate inflammation and motility	Limited SAGD data; protocol variability	Indirect clinical and early sepsis data

Current therapies are mainly supportive, addressing specific aspects of gastrointestinal function, whereas neuromodulation approaches target the underlying neuroimmune and autonomic dysfunctions contributing to SAGD. Despite promising results, neuromodulation strategies like taVNS are still investigational and require further clinical validation.

## 4 Emerging therapeutic strategies: neuroimmune modulation and vagus nerve–mediated regulation

### 4.1 Gut–brain axis disruption and vagus nerve–mediated neuroimmune regulation

The gut–brain axis constitutes a bidirectional communication network that integrates neural, immune, endocrine, and metabolic signaling pathways and plays a central role in maintaining gastrointestinal and systemic homeostasis (53, 81–83). Building on the inflammatory and neuroimmune mechanisms outlined above, disruption of this axis further amplifies gastrointestinal dysfunction in sepsis-associated gastrointestinal dysfunction (SAGD) (81–83). As discussed above, autonomic imbalance—often reflected by altered vagal modulation and sympathetic predominance, particularly during acute sepsis—is a hallmark of sepsis and has been associated with disease severity and adverse outcomes, frequently reflected by impaired heart rate variability (13, 59, 84).

Beyond its role in cardiovascular regulation, vagal signaling exerts important modulatory effects on both immune responses and gastrointestinal function (14, 53, 81–83). Mechanistically, vagal regulation of inflammation is mediated through the cholinergic anti-inflammatory pathway, a neuroimmune reflex linking central autonomic control with peripheral immune responses. Through coordinated afferent and efferent signaling, this pathway modulates inflammatory activity and contributes to the regulation of systemic cytokine responses, as described in vagal anti-inflammatory pathways above. These processes provide a functional link between vagal activity, systemic inflammation, and gastrointestinal dysfunction (14, 33, 85–88).

At the same time, vagal signaling also plays a key role in regulating gastrointestinal motility, secretion, and enteric neural coordination through its interactions with the enteric nervous system (ENS). Altered vagal signaling may therefore contribute not only to dysregulated inflammatory responses but also to dysmotility, impaired gastric emptying, and barrier dysfunction observed in SAGD (81–83, 88, 89). Taken together, these observations suggest that vagus nerve dysfunction represents a central mechanistic link connecting inflammation, neuroimmune imbalance, and gastrointestinal dysfunction, and thus a biologically relevant therapeutic target. These integrated neuroimmune effects may be particularly relevant in SAGD, where autonomic dysregulation, systemic inflammation, and intestinal dysfunction interact through the gut–brain axis (14, 53, 81–83, 89).

### 4.2 Neuromodulation of the vagus nerve: mechanistic and experimental evidence

Given the central role of vagal signaling in neuroimmune regulation, neuromodulation strategies targeting the vagus nerve have increasingly attracted attention as potential therapeutic approaches (53, 83, 88). Implantable vagus nerve stimulation (VNS) has demonstrated clear anti-inflammatory effects in experimental models, including attenuation of tumor necrosis factor (TNF) release, reduction of systemic cytokine responses, and improved survival in preclinical models of sepsis (16, 33, 90). These effects are consistent with engagement of vagal anti-inflammatory pathways described above (16, 33, 87, 90). However, it is important to note that these findings are largely derived from controlled animal models or endotoxemia settings and may not fully reflect the complexity of human sepsis. In clinical settings, multiple factors—including comorbidities, infection source and control, antimicrobial therapy, hemodynamic instability, and organ support—may substantially influence treatment responses and limit direct translational applicability.

Beyond its immunomodulatory effects, vagal stimulation has also been shown to influence gastrointestinal physiology by modulating enteric neuronal activity, smooth muscle contractility, and secretory processes, thereby contributing to the restoration of gastrointestinal function (15, 53, 83, 88). In addition, experimental evidence suggests that vagal activation may affect intestinal barrier integrity and epithelial permeability, although the underlying mechanisms remain not fully understood (15, 83).

However, the invasive nature of implantable VNS limits its clinical applicability in critically ill patients, particularly in the acute setting of sepsis, where rapid, flexible, and low-risk interventions are needed (83, 88). Importantly, evidence supporting vagus nerve–targeted neuromodulation in this context comes from multiple sources, including experimental sepsis models, studies of inflammatory diseases, and research in functional gastrointestinal disorders (53, 83, 87, 88). While these findings collectively support a strong mechanistic rationale, direct clinical evidence specifically addressing SAGD remains limited, and this distinction between mechanistic plausibility and clinical validation should be carefully considered when interpreting the translational potential of neuromodulation strategies (83, 88).

### 4.3 Translational potential of taVNS in sepsis-associated gastrointestinal dysfunction

Transcutaneous auricular vagus nerve stimulation (taVNS) has emerged as a non-invasive and clinically feasible alternative to implantable VNS, targeting the auricular branch of the vagus nerve. Through activation of afferent fibers projecting to the nucleus tractus solitarius and subsequent engagement of central autonomic circuits, taVNS can influence both afferent and efferent vagal pathways, thereby modulating downstream neuroimmune responses. This central integration enables taVNS to affect multiple physiological systems, including immune regulation, autonomic balance, and gastrointestinal function (18, 53, 83, 91).

From a mechanistic perspective, taVNS may act on several key pathophysiological processes implicated in SAGD. First, engagement of vagal anti-inflammatory pathways may attenuate systemic inflammatory responses, although this mechanism remains to be directly validated in SAGD-specific clinical settings (53, 92–94). Second, enhancement of vagal tone may help re-establish autonomic balance, potentially improving coordination between sympathetic and parasympathetic activity (92, 95, 96). Third, modulation of vagal–enteric interactions may improve gastrointestinal motility, gastric emptying, and coordination within the ENS (19, 91, 97, 98). Fourth, indirect effects on intestinal microcirculation, epithelial integrity, and mucosal barrier function have been proposed, although these mechanisms are not yet fully defined (19, 53, 91, 98).

Emerging evidence from experimental studies and clinical research in related conditions, such as functional gastrointestinal disorders, postoperative ileus, and inflammatory diseases, supports the capacity of taVNS to influence both inflammatory signaling and gastrointestinal function (19, 93, 94, 97, 98). In addition, improvements in autonomic markers, including heart rate variability, have been observed in some clinical settings, suggesting effective engagement of vagal pathways (92, 95, 96). However, direct clinical evidence in patients with SAGD remains limited, and extrapolation from these conditions requires cautious interpretation (19, 91, 97, 98). Importantly, existing clinical studies of taVNS have generally been conducted in populations other than septic patients with gastrointestinal dysfunction, and many have focused on physiological or biomarker-based outcomes rather than validated SAGD-specific endpoints. As a result, current evidence should be regarded as hypothesis-generating rather than confirmatory.

From a translational perspective, the potential applicability of taVNS in critically ill patients depends not only on its mechanistic plausibility but also on its feasibility within the intensive care unit (ICU) environment. As a non-invasive modality, taVNS offers practical advantages over implantable VNS, including ease of use, reversibility, and a favorable safety profile. These features may allow for repeated or continuous stimulation without the risks associated with surgical implantation (18, 53, 83, 91). However, several practical considerations remain. The presence of sedation, mechanical ventilation, and hemodynamic instability may affect both the delivery of stimulation and the interpretation of physiological responses (53, 91). In addition, patient-related factors, such as skin integrity at the stimulation site and the presence of arrhythmias or implanted devices, may influence feasibility and safety (18, 53, 83, 91).

Another important challenge relates to the lack of standardized stimulation protocols. Current studies vary considerably in terms of stimulation site (e.g., cymba conchae vs. tragus), frequency, pulse width, current intensity, and duration of stimulation, making comparisons across studies difficult (18, 92, 96). The optimal timing of intervention in sepsis—whether early during systemic inflammatory activation or later during established organ dysfunction—also remains uncertain (53, 91). Furthermore, dose–response relationships and treatment duration have not been clearly defined, particularly in critically ill populations (18, 92, 96).

The selection of appropriate clinical and mechanistic endpoints represents an additional challenge for translational research. Potential clinical endpoints may include measures of gastrointestinal function, such as feeding tolerance, gastric residual volumes, time to achieve target enteral nutrition, and resolution of ileus. Mechanistic endpoints may include inflammatory biomarkers, indices of intestinal barrier function, and measures of autonomic activity such as heart rate variability (10, 19, 53, 91, 92, 95, 96, 98). Integrating these endpoints in future studies may provide a more comprehensive assessment of the effects of taVNS (53, 91, 92).

At present, taVNS should therefore be regarded as a hypothesis-driven, mechanism-based adjunctive approach rather than an established therapy for SAGD (53, 91). Further well-designed studies are needed to determine its clinical efficacy, optimize stimulation parameters, and define its role within multimodal treatment strategies for sepsis-associated gastrointestinal dysfunction (18, 19, 53, 91, 92, 97, 98).

### 4.4 Knowledge gaps and future directions

Although the mechanistic rationale for vagus nerve–targeted neuromodulation in SAGD is biologically plausible, the current evidence base remains limited and should be interpreted with caution. Much of the available evidence derives from experimental sepsis models, studies of intestinal inflammation, functional gastrointestinal disorders, or general critical illness rather than from patients with SAGD specifically. Therefore, extrapolation to SAGD requires caution. In particular, the heterogeneity of septic patients and the lack of standardized, clinically meaningful endpoints represent major barriers to translation into clinical practice.

#### 4.4.1 Patient selection and stratification

Septic patients are highly heterogeneous in terms of infection source, disease severity, hemodynamic status, sedation exposure, nutritional tolerance, and baseline autonomic function. This heterogeneity complicates interpretation of treatment effects and highlights the need for improved patient selection strategies. Future studies should aim to identify patient subgroups most likely to benefit from neuromodulation based on clinical phenotype, autonomic function, or biomarker profiles (53, 99).

#### 4.4.2 Optimization of stimulation parameters

A major limitation of current research is the lack of standardized stimulation protocols. Existing studies vary substantially in stimulation site, frequency, intensity, treatment duration, and timing relative to disease onset, making comparisons difficult and limiting reproducibility. Systematic evaluation of dose–response relationships and optimization of stimulation parameters will be essential to define clinically applicable protocols (18, 99, 100).

#### 4.4.3 Clinically meaningful endpoints and biomarkers

Many existing studies rely on surrogate endpoints, such as cytokine levels, heart rate variability, gastrointestinal motility indices, or barrier-related biomarkers, whereas clinically meaningful SAGD-specific outcomes remain insufficiently standardized. Reliable biomarkers for assessing vagal activity and treatment response also require further validation (99, 101). Future studies should integrate mechanistic biomarkers with clinically relevant endpoints—such as feeding tolerance, gastrointestinal motility, and clinical outcomes—to enable robust assessment of therapeutic efficacy (99, 101, 102).

Despite these challenges, continued investigation into vagus nerve–targeted neuromodulation may help bridge the gap between mechanistic insights and clinical application. Addressing these key issues will be essential for translating neuromodulation strategies into clinically effective interventions and for clarifying the role of taVNS in the management of SAGD (18, 53, 99, 100). The proposed mechanisms and translational considerations of taVNS in SAGD are summarized in Table 2.

**TABLE 2:** Mechanistic pathways and translational considerations of taVNS in SAGD.

Domain	Mechanism	Relevance to SAGD	Evidence	Limitations
Neuroimmune	Vagal anti-inflammatory pathways	↓ systemic inflammation	Preclinical; pilot sepsis data	No SAGD-specific trials
Autonomic	Sympathovagal modulation	Improves motility; gut–brain signaling	HRV-based studies	HRV confounded in ICU
Motility	Vagal–enteric interaction	↑ gastric emptying; ↓ dysmotility	Healthy + FGID studies	No septic validation
Barrier	Indirect anti-inflammatory effects	May improve permeability	Preclinical rationale	Clinical evidence lacking
Microcirculation	Autonomic vascular modulation	Potential perfusion improvement	Hypothesis-driven	No direct evidence
Clinical use	Non-invasive neuromodulation	Feasible in ICU	Early clinical data	Protocols not standardized
Translation	Integrative effects	Links inflammation, motility, autonomic function	Mixed evidence	Requires RCTs; endpoints unclear

Although vagus nerve stimulation is biologically plausible for modulating neuroimmune and autonomic pathways in SAGD, direct clinical evidence remains limited. Most supporting data are derived from preclinical studies, indirect clinical evidence, or related conditions, and further research is needed to validate its therapeutic potential in SAGD.

## 5 Conclusion

Sepsis-associated gastrointestinal dysfunction (SAGD) is a multifactorial condition arising from the interplay of systemic inflammation, intestinal barrier disruption, microcirculatory impairment, and neuroimmune dysregulation, rather than an isolated gastrointestinal disorder. Current therapeutic strategies remain largely supportive and do not adequately address these interconnected mechanisms, underscoring the need for approaches more closely aligned with underlying pathophysiology. The vagus nerve is increasingly recognized as a key regulator within the gut–brain axis, linking neural and immune responses. Neuromodulation strategies targeting vagal pathways, particularly transcutaneous auricular vagus nerve stimulation (taVNS), represent a biologically plausible and clinically feasible investigational approach. However, current evidence remains largely indirect, and direct SAGD-specific clinical validation is still limited. Key challenges—including patient heterogeneity, optimization of stimulation parameters, and the development of clinically meaningful endpoints—must be addressed to advance translation. Further well-designed studies are required to clarify the therapeutic role of vagus nerve–targeted neuromodulation in SAGD and to bridge the gap between mechanistic insights and clinical application.
